# Aquaphotomics monitoring of strawberry fruit during cold storage – A comparison of two cooling systems

**DOI:** 10.3389/fnut.2022.1058173

**Published:** 2022-12-09

**Authors:** Jelena Muncan, Sukritta Anantawittayanon, Tetsuya Furuta, Toshiya Kaneko, Roumiana Tsenkova

**Affiliations:** ^1^Aquaphotomics Research Department, Graduate School of Agricultural Science, Kobe University, Kobe, Japan; ^2^Nichiei Intec Co., Ltd., Tokyo, Japan

**Keywords:** strawberry, cold storage, aquaphotomics, near infrared spectroscopy, water, water molecular structure, electric field (EF), monitoring

## Abstract

The objective of this study was to use aquaphotomics and near-infrared (NIR) spectroscopy to follow the changes in strawberries during cold storage in the refrigerator with an electric field generator (supercooling fridge, SCF) and without it (control fridge, CF). The NIR spectra of strawberries stored in these refrigerators were collected over the course of 15 days using a portable mini spectrometer and their weight was measured daily. The spectral data in the region of the first overtone of water (1,300–1,600 nm) were analyzed using aquaphotomics multivariate analysis. The results showed a decrease in weight loss of strawberries, but the loss of weight was significantly lower in SCF, compared to the CF. The reduction of weight loss due to exposure to an electric field was comparable to the use of coatings. The aquaphotomics analysis showed that the NIR spectra adequately captured changes in the fruit over the storage period, and that it is possible to predict how long the fruit spent in storage, regardless of the storage type. During aquaphotomics analysis, 19 water absorbance bands were found to be consistently repeating and to have importance for the description of changes in strawberries during cold storage. These bands defined the water spectral pattern (WASP), multidimensional biomarker that was used for the description of the state and dynamics of water in strawberries during time spent in storage. Comparison of WASPs of strawberries in CF and SCF showed that exposure to an electric field leads to a delay in ripening by around 3 days. This was evidenced by the increased amount of structural, strongly bound water and vapor-like trapped water in the strawberries stored in SCF. This particular state of water in strawberries stored in SCF was related to the hardening of the strawberry skin and prevention of moisture loss, in agreement with the results of significantly decreased weight loss.

## Introduction

Strawberry is a well-known fruit consumed in both fresh and processed forms, not only because of its unique taste and fragrance but also its beneficial health properties. Strawberries contain numerous nutrients, vitamins, and minerals and plenty of compounds with biological effects, such as anthocyanins, flavonoids, and phenolic acids, which are shown to have anti-oxidative, anti-inflammatory, antihypertensive, anticancer, and anti-neurodegenerative properties (1–3).

Strawberry is a non-climacteric fruit, one of the most perishable ones, with a short post-harvest life, prone to rapid spoilage, mechanical injury, softening, and infections by several pathogens (2, 4), which necessitates special care during transport and storage to maintain a stable supply throughout the year. To slow down the metabolism and reduce deterioration, immediately after harvesting and prior to transport or storage, strawberries are cooled to 0°C (5, 6), followed by continuous storage at low-temperatures (0–4°C) (7) to control fruit respiration and extend the shelf-life which is usually around 5 days (8). In addition to the prevalent techniques of controlling decay, which are based on rapid cooling after harvest and storage at low temperature, other techniques are investigated to maintain quality, such as heat treatment, smart packaging materials, edible coatings, modified storage atmosphere, irradiation, and others (5, 8–19). All the physical and chemical preservation techniques are aimed to control the available oxygen and moisture transfer, thereby decreasing the respiration and transpiration trend and reducing weight loss, shrinkage, and microbial activity (13, 20, 21).

Packaging and coating benefits are, however, dependent on consumers’ behavior, and several studies showed that a large percentage of consumers (at least 50%, up to 89% in some cases) remove the coating or open the package, not recognizing that it has a function of preserving the food quality (22–24). In addition, as several studies showed, most of the current preservation strategies are money and time-consuming, while fruit still experiences changes in color or flavor (14, 25–27). The application of electromagnetic fields in cooling systems is one of the novel strategies explored with the aim to develop better storage conditions for agricultural products. Research studies have shown that electromagnetic treatment helps the biological systems activate their defensive reactions, leading to an increase in the ability to repair the physical damages and benefit preservation (28–32).

Most fruits, including strawberries, have high water content per weight. In strawberries, about 90% of their weight is water. Water is a strong absorber of light - it absorbs over the entire electromagnetic spectrum, but in the near-infrared (NIR) spectral region 700–2,500 nm, the absorbance of water, especially compared to infrared light, is much smaller, making spectroscopic characterization possible for samples with high water content (33). Near-infrared (NIR) spectroscopy (NIRS) is a widely used non-destructive method that allows the acquisition of data without damaging the samples.

The primary features of the NIR spectrum of water, or any sample with high water content, are two prominent bands, called the first overtone of water and combination band, with several smaller bands due to higher overtones and combinations (34). Owing to the employment of many chemometrics and data analysis techniques, it is now well-recognized that these broad features are composed of many specific water absorbance bands that can be assigned to specific water molecular species (conformations). This new lens to the bio-system investigation was first recognized by a young scientific discipline, aquaphotomics, established by Prof. Dr. Roumiana Tsenkova in 2005 (35). The NIR aquaphotomics takes advantage of non-destructive NIRS measurements and ground-breaking knowledge of water–light interaction for the development of the non-destructive, integrative analysis of intact biological systems (34). This concept opens a novel, dynamic, non-invasive way of biosystems monitoring.

This research aimed to utilize aquaphotomic NIRS for monitoring during cold storage and better understanding of the mechanisms for maintaining freshness, choosing the strawberry fruit as a fruit-model system. The combination of NIRS and aquaphotomics has been already used to monitor aqueous and biological systems non-invasively for various purposes, such as evaluation of fresh and processed fruits and vegetables quality (36–42), milk quality (43, 44), viral infections in plants, bacterial cultures (45, 46) and fermentation (47–49), and physiological monitoring and diagnostics in higher organisms (50–55). The second aim of the investigation was to compare the effects of different cooling technologies on the preservation of strawberries. The applications of aquaphotomics in the monitoring during storage and estimation of freshness and shelf-life have only recently been explored (41, 56). This study employed two different cooling technologies. The first cooling system was based on conventional refrigeration technology, while the second one, employed exactly the same system but with the addition of an electric field generation device. The effects of different cooling systems were explored using non-destructive measurements by near-infrared spectroscopy (NIRS) and evaluated using an innovative aquaphotomics approach, which provides information about the molecular structure of water in the strawberries and connects it with their system functionality. The aquaphotomics analysis was focused on the spectra of strawberries collected in the NIR range 1,300–1,600 nm, the first overtone of water symmetric and asymmetric stretching vibrations. To this date, this range of spectra is considered the best for the understanding of the water molecular structure in various bio-aqueous systems in relation to their observed functionality, which is well-documented in many literature sources (35, 57–60).

To the authors’ knowledge, only one research study used a similar approach to monitor changes during the storage of fresh produce (41), but this study went even further with the added novelty of exploring the differences in stored fruit depending on the employed cooling technology. While there are scientific studies concerned with the exploration of how novel cooling technologies affect food during storage or extend shelf life, the studies which explain the underlying mechanisms behind the shelf-life extension are still scarce. The application of aquaphotomics and in-depth investigation of water molecular structure in fruit during storage and how it changes with the time spent in storage, described in minute details, as this work will show, is a pioneering one and might set the direction of future research in this area. Interpretation of the found differences within the framework of known functionality of different water molecular species could serve as a basis for improved refrigeration, maintaining freshness, and prolonged shelf life. This information will provide novel possibilities for the development of non-destructive food monitoring technologies and a better understanding of postharvest food preservation mechanisms.

## Materials and methods

### Samples and storage conditions

Strawberries (*Fragaria L.*) of the ‘New Harumi’ cultivar were purchased in the local supermarket in Hyogo prefecture in Japan. After the transport to laboratory, the fruits were screened for uniformity, defects, and damage, and without washing or any other treatment, randomly distributed on the paper plates, eight strawberries per plate. Each individual fruit was assigned with the number label on the paper to provide identification of each biological replicate. The samples were then stored in different cooling system environments, 24 strawberries (three plates) per each storage condition – in the commercially available refrigerator (Model no. YRC-080RM2, Nichiei Intec Co., Ltd, referred to as a “Control Fridge: CF”), and also commercially available refrigerator, which employs electric field (Super Cooling Plant, Nichiei Intec Co., Ltd., referred to as a “Super Cooling Fridge: SCF” (61). The sinusoidal waveform in the frequency range of 48–62 Hz is used to form an electric field; the field is static with a strength of 3,000 V.

The nominal working temperature of both refrigerators was set to the same value of *T* = 0°C with a relative humidity of 90%. The samples were stored without any cover, packaging, or coating, in order to examine only the effects of different cooling systems, simulating the typical scenario of how the majority of consumers would behave.

### Weight loss monitoring

The weight of each strawberry sample was measured immediately before the spectral acquisition for each fruit each day during the period of monitoring. Weight loss rate was calculated from the weight difference compared to the first measurement, when the storage period started, for each individual strawberry, according to the following equation:


(1) $$Weightlossrate\left(\%\right)=\frac{\left(W_{f}-W_{s}\right)}{W_{f}}\times 100,$$

Where *W_f_* represents the weight of a particular sample before storage (fresh) and *W_s_* represents the weight of the sample on the respective day of storage.

To explore the dynamic changes in weight loss, the daily weight loss was also calculated. Daily weight loss represents the weight difference between a certain day of storage compared to the weight measured on the previous day, which is plotted as a function of time and shows the dynamic weight change in the strawberries.

### Near infrared spectral acquisition

Spectral acquisition was performed using a portable, hand-held MicroNIR instrument (Viavi Solutions, Santa Rosa, CA, USA) in the spectral range of 908–1,670 nm, with approximately 7 nm resolution step.

**FIGURE 1:**
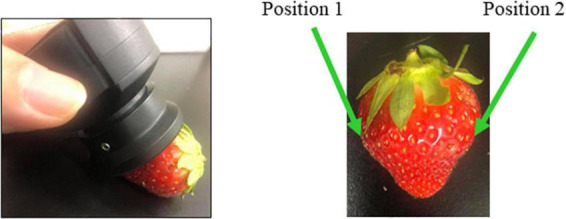
Acquisition of strawberry spectra using hand-held MicroNIR spectrometer was performed on two measurement positions.

The spectra were collected from 2 positions, front and back (Figure 1), per sample, with three consecutive measurements, for 14 days of the storage duration. The measurements were performed at 3.00 PM every day. The spectra collected on the first day of experiment, prior to putting the strawberries in the cold storage, are labeled as “day 0;” therefore, these are the spectra of fresh strawberries. The spectra collected afterward are labeled according to the number of days spent in cold storage, such as “day 1” and “day 2” finishing with “day 14”. In total, 2,160 spectra (15 days × 24 strawberries × 2 positions × 3 consecutive measurements) were collected from each storage condition group.

### Data analysis

#### Statistical analysis of weight loss

A paired samples *t*-test assuming equal variance at 95% confidence level was performed to confirm the significant difference in the %weight loss rate and daily weight loss between the two different cooling storage systems during the first 7 days of the storage. The statistical analysis was performed using Microsoft Excel (Microsoft, Redmond, WA, USA).

#### Multivariate analysis

Multivariate analysis of the water spectra was performed using Pirouette software v4.5 (Infometrix Inc. USA). Standard normal variate (SNV) transformation (62) was applied as a pre-processing treatment to eliminate the baseline differences among the spectra before calculating the difference spectra. In all other instances, the analysis was performed using raw spectra to introduce all available information to the model.

The difference spectra were calculated by subtracting the averaged spectrum of SNV-transformed spectra acquired on the first day of the storage [S_day (1)_], from each of the averaged SNV-transformed spectra on subsequent days [S_day(n)_] to see the spectral changes during the storage, as given by the following equation:


(2) $$Difference\,spectra=avg\,\left(S_{day\left(n\right)}\right)-avg\,\left(S_{day\left(1\right)}\right).$$

Soft independent modeling of class analogies (SIMCA) analysis, a supervised classification method (63), was performed with the objective of discrimination between different days of storage [class variable – duration of storage (days)]. This algorithm employs principal components analysis of spectra for the construction of mathematical models for each sample group (class: storage day) with confidence limits. Interclass distances are calculated using between-class residuals, and variable importance is determined by comparing the average residual variance of each class to all classes and the residual variance of all classes to themselves. Variable importance, known as discriminating power, was used to find variables (wavelengths) with the highest contribution to discrimination between classes. The analysis was performed using mean-centered spectra, and the SIMCA models were developed separately for each cooling system.

Partial least squares regression (PLSR) analysis (64) was performed to explore spectral changes as the function of the storage duration (number of storage days). The models were developed separately for each different cooling system, and the water spectral data were mean-centered prior to regression modeling using the number of days of storage as a dependent variable. The analysis was applied twice to investigate changes during 4 and during 14 days of storage, according to the changing trends shown in the difference spectra. The validation was performed using stepwise exclusion of six spectra in each iteration. The accuracy of the PLSR models was evaluated by determining the coefficient of determination of calibration (R_*c*_^2^), standard error of calibration (SEC), coefficient of determination of cross validation (R_*cv*_^2^), and standard error of cross-validation (SECV). The optimum number of latent variables (LV) was determined based on the lowest SEC and lowest SECV, for calibration and cross-validation, respectively. The maximum number of LVs (factors) included in all models was limited to 15 in order to include as much as possible information but based on visual inspection of regression vectors for any appearance of “jaggedness” to prevent overfitting (65).

#### Aquagrams

Aquagrams (66, 67) were used to visualize the water spectral pattern in the strawberry flesh and how it changes along with time and depending whether the strawberries were stored in controlled or supercooling fridge. The representative water absorbance bands that were used as radial axes on aquagram, were selected from all the analysis results, following the procedure described elsewhere (67, 68) to ensure their importance and consistency. There are several ways to create aquagrams, depending on the purpose of the investigation (67). In this study, the aquagrams were calculated according to the procedure for calculating classical aquagram (67). In brief, the SNV – transformed and normalized absorbance values were averaged for each day, for SCF and CF conditions separately, and the values calculated for the first day of storage were subtracted. This calculated difference in absorbance was plotted at the found representative water absorbance bands to illustrate the changes in strawberries each day and depending on the storage conditions. The aquagram display was created in two different ways, first to allow the comparison of the storage conditions in general, and second, to provide better insight into the differences between the strawberries stored in different conditions on day-to-day basis.

## Results and discussion

### Weight loss of the strawberries during storage

Although the values were slightly different between individual strawberries, due to the natural variety of the biological replicates, the weight of the strawberries during storage, on average, linearly decreased with the progress of storage time irrespective of the applied cooling system. The weight was monitored for 14 days; however, here, only the results of weight change after 7 days will be presented, to allow comparison with most of the available literature sources.

**FIGURE 2:**
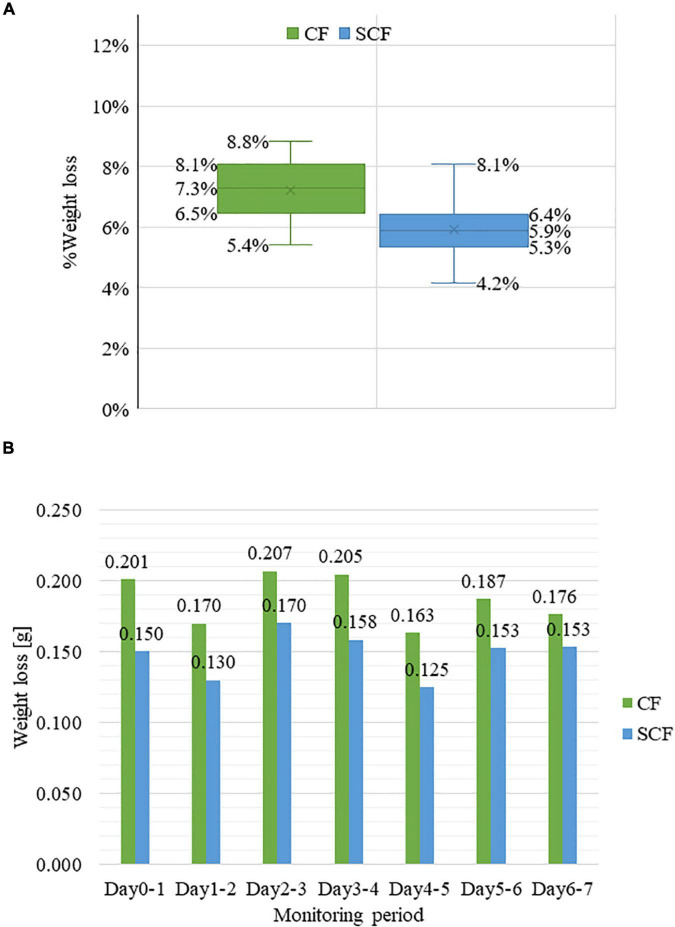
Weight loss during the storage. **(A)** % weight loss of strawberry samples in control fridge (CF) and supercooling fridge (SCF) after 7 days of storage **(B)** the daily weight change of the strawberry samples in CF and SCF conditions within 7 days.

There was a significant difference in the weight loss rate of strawberries stored in the control CF (mean M = 7.3%, standard deviation SD = 0.009737) and supercooling fridge SCF (mean M = 5.9%, standard deviation SD = 0.009964) [*t*(df) = 3.3342, *p* = 0.000849; Figure 2A] after 7 days of storage. The weight loss during this period was shown to be fluctuating on a day-by-day basis (Figure 2B) but mainly followed the linear trend as the function of storage days, in agreement with many previous studies (5, 10, 69). Despite the similar pattern in weight loss from day to day, the weight loss of strawberries in SCF was each day smaller in comparison with CF.

The loss of weight in strawberries is associated with the respiration rate and evaporation of water through the fruit surface; the rapid loss of water is the major factor associated with the perishability of fruits, leading to shrinkage and deterioration (70–72). The observed conservation of weight in strawberries in SCF indicates that the exposure to the electric field might be delaying water loss, probably through prevention of the water evaporation. The weight loss of around 6% after 7 days in cold storage is comparable to the influence of coatings which form semi-permeable layer on fruit surface and act as protective barrier to reduce transpiration (7, 73, 74). For example, using chitosan coatings with different molecular weight was reported to limit the weight loss in strawberries to 5.28–5.66% after 14 days of storage at 4°C with the relative humidity of 85% (69). Gelatin coating resulted in 5.26% weight loss after 12 days of storage at 4°C and the relative humidity of 95% (10).

Super cooling fridge showed more potential to limit the water loss in the strawberry samples with a significant difference. Since water loss in strawberry is actually related to respiration and transpiration, this finding suggests that that presence of electric field in SCF influences these major cellular processes (13, 75). Controlling relative humidity in storage in combination with using electric field exposure seems to be promising direction to further limit the water loss which will be investigated in the next phase of this research.

### Difference spectra and the changes in the water matrix of fruit flesh during storage

**FIGURE 3:**
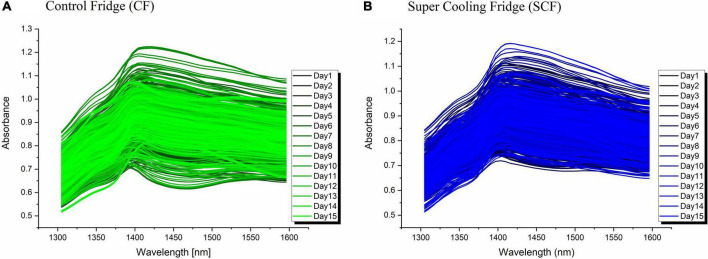
Raw absorbance spectra of strawberries in **(A)** control fridge (CF); **(B)** Super Cooling Fridge (SCF).

The raw absorbance spectra of strawberries collected over the course of 15 days is presented in Figure 3A for strawberries in CF and Figure 3B for SCF conditions, respectively. Prior to spectral subtraction, the spectra from both groups were corrected for baseline differences using SNV transformation.

**FIGURE 4:**
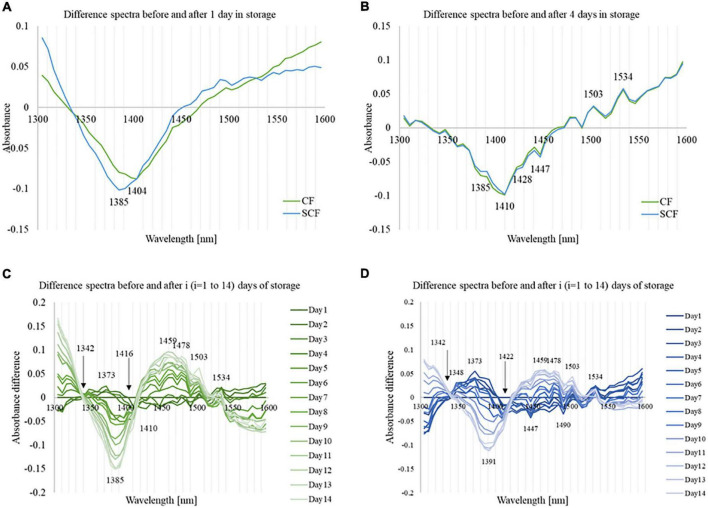
Difference spectra of the strawberry [Day_(i)_ – Day_(1)_] at different times during storage. **(A)** The difference spectra from day 0 to day 1 showing the impact of low-temperature storage and different cooling systems. **(B)** The difference spectra from day 0 to day 4 showing initial changes during storage and differences between the cooling systems. **(C)** The difference spectra of the strawberry [Day_(i)_ – Day_(1)_] during the storage in control fridge (CF). **(D)** The difference spectra of the strawberry [Day_(i)_ – Day_(1)_] during the storage in supercooling fridge (SCF).

The difference between spectra measured when the samples were just divided into two different groups and assigned to different storage conditions (day 0), and the spectra measured after the samples spent 1 day of storage (day 1) in CF and SCF, respectively, are shown in Figure 4A. The spectra for each group were averaged, and the difference spectra calculated by subtracting average spectrum at day 0 from average spectrum at day 1. The difference spectra reveal decreased absorbance with the minimum in 1,350–1,450 nm regions after 1 day of cold storage. The entire region corresponds to absorption of weakly hydrogen-bonded water, which encompasses proton hydrates, water hydration shells, and water vapor (56) among other water species, indicating the loss of water from fruit through transpiration, in agreement with what was observed in weight loss measurements.

The change for strawberries in SCF showed the minimum absorbance peak located at 1,385 nm (water hydration shell) (66). In comparison, the change in absorbance was less intense for strawberries stored in CF in the region 1,350–1,400 nm, but slightly more in 1,400–1,450 nm, with the minimum located at 1,404 nm (free water) (66). These results revealed different nature of changes in the water molecular structure of strawberry flesh due to the cooling in CF and SCF.

The differences in effects of CF and SCF started to decrease after the strawberries spent 4 days in storage, as can be seen from the difference spectra between the 4th and 1st day of storage of strawberries in the CF and SCF fridge given in Figure 4B. Interestingly, the subtracted spectra in both groups had very similar spectral patterns based mostly on the same water absorbance bands. It is important to note here that the subtracted spectra were not spectra collected prior to cold storage (day 0); therefore, the difference spectra reflect only the changes in strawberries due to the time spent in cold storage.

The value of absorbance in the region 1,350–1,450 nm stayed on the negative side, showing that the continuous loss of weakly hydrogen-bonded water is a common feature for both CF and SCF. The subtle difference between the storage conditions could be seen in the absorbance region around 1,380–1,410 nm, where absorbance bands of water hydration shells (1,380–1,388 nm), trapped water (1,396–1,403 nm) and free water molecules (1,404–1,418 nm) are located (66, 76). The decrease in absorbance of these water species, as well as the weight loss, is more pronounced for strawberries stored in CF.

The daily difference spectra during the storage of strawberries in CF and SCF when compared to day 1 are presented in Figures 4C,D, respectively. The difference spectra were calculated compared to day 1, to investigate how the spectra are change each day during cold storage and if there is a difference between the cooling systems. In the first 3 days, the spectra revealed increasing trend of the absorbance in 1,340–1,400 nm region, with the highest peak at 1,373 nm (proton hydrates, one of the absorbance bands of water vapor) (44, 56, 59). The absorbance in that part reached the maximum value on the 4th day of the storage, and then declined. CF and SCF provided similar results, while the maximum of the absorbance change at 1,373 nm is larger in the SCF group. The increased absorbance in this region suggests increase in the amount of gaseous phase of water within the fruit, which is characterized by highly mobile water species that can be exchanged with the environment. These water species are highly related to the water activity (56), too.

After 5th day, the absorbance in 1,340–1,420 nm of the CF group drop rapidly, while the change in SCF condition occurred gradually. The decreased absorbance at 1,340–1,420 nm had main negative peak located at 1,385 nm for CF, and at 1,391 nm for SCF conditions, respectively, which suggests controlled dehydration for the SCF group. The absorbance in the region 1,434–1,540 nm showed only minor changes until the 4th day of storage, then increased, with the major positive peaks located at 1,459, 1,478, 1,503, and 1,534 nm, and the main negative peaks at 1,447 and 1,490 nm. The absorbance at wavelengths above 1,534 nm was intensively decreased in the case of CF after 5 days of storage, while for SCF, the decrease was very gradual over the storage period and leveled in the last days of monitoring. Taking this into consideration, it seems that beginning from day 5, the changes in spectra of strawberries become very pronounced which is consistent with the reports that the shelf life of strawberries is around 5 days in cold storage (8).

It is interesting to point out that at two locations in the spectra there was a minimum change in absorbance throughout the storage, for both cold storage types (indicated by arrows in Figures 4C,D). The first one is located at around 1,342 nm, while the second one was located at 1,416 nm in the case of the CF, while at 1,422 nm in the case of SCF.

All peaks that were found in the analysis so far are located within the first overtone of water symmetric and asymmetric stretching vibrations, and with the exception of 1,534 nm, all are within the ranges of, already discovered in other systems, Water Matrix Coordinates (WAMACS), absorbance bands of particular water molecular conformations (66). More detailed assignment of these bands and interpretation from the aspect of water molecular structure will be provided later, in the 3.5 Choosing WAMACS and 3.6 “Aquagrams” section.

### Soft independent modeling of class analogies analysis of the storage time classification

Soft independent modeling of class analogies analysis was performed with the objective of classifying spectra of strawberries according to the day of spectral acquisition, therefore, developing a model for prediction of how many days the strawberries were stored according to their spectra. The analysis was repeated three times, to explore the possibility of prediction for different lengths of storage time and for exploration of the most influential variables in SIMCA models. These variables, as given by discriminative power vector, can provide insight into the changes in water matrix of the fruit during storage as a function of the storage time and also as a function of different cooling system technology.

For all three SIMCA analyses, performed for the periods of 4, 8, and all 14 days, the results showed good accuracy of classification between the storage days and satisfying interclass distance.

In the case of SIMCA models developed based on the spectra from all 14 days, the classification accuracy was exceptionally high for both CF (97.87% accuracy) and SCF (99.26% accuracy) storage conditions, showing excellent possibilities of using NIR spectra for prediction of the freshness of strawberries, i.e., of predicting how many days the fruit spent in cold storage.

**FIGURE 5:**
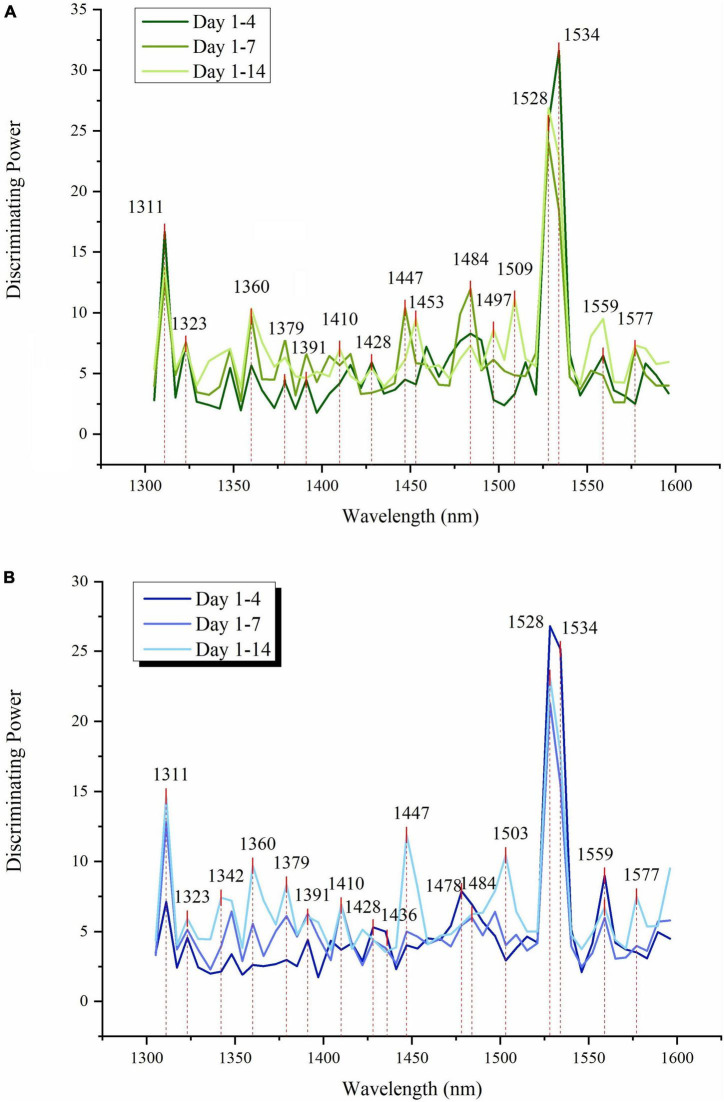
Soft independent modeling of class analogies (SIMCA) discriminating powers of absorbance bands show the importance of individual variables in NIR spectra for discrimination between the storage days of strawberries during 4, 8, and 15 days of storage **(A)** SIMCA results for CF group **(B)** SIMCA results for SCF group.

The values of interclass distance between storage days was larger than 1, which can be considered good, according to the reports that less than 0.8 would be a small difference (49). The discriminating powers of SIMCA models provided in Figure 5 allow comparison of influential variables over time (since models were built for 4, 7, and 14 days) and depending on employed cooling technology - CF (Figure 5A) and SCF (Figure 5B), respectively. Discriminating power shows which absorbance bands contributed most to the successful discrimination between storage days.

The absorbance band with the highest discriminating power, representing an essential feature for all classification models, was found located at 1,528–1,534 nm for both CF and SCF conditions. In the classification model using 14 days of data, the discriminating powers of SIMCA models for CF and SCF show the common peaks at 1,311 and 1,360 nm, while 1,447 nm was more prominent in the SCF discriminating powers. SIMCA analysis results for different time periods (4, 7, and 14 days) revealed that the absorbance bands at 1,311 nm and 1,528–1,540 nm are always the variables with the highest discriminating power, describing where in spectra are the largest differences with respect to the day of the storage.

The discriminating power at 1,311 nm shows an increase along with time for SCF models but decreases for CF SIMCA models. The band located at 1,447 nm showed higher discriminating power during the first 7 days of storage for strawberries in CF, but it had highest power for 14 days of storage data when it comes to the SCF group. For CF data, the discriminating power of 1,534 nm surpassed 1,528 nm in the model using 4 days of data, but for the longer period, the discriminating power of 1,528 became higher than 1,534 nm, while in SCF 1,528 nm always had higher discriminating power than 1,534 nm regardless of the length of time.

The interesting difference between the discriminating powers between CF and SCF group is the pair of absorbance bands located at 1,360 and 1,379 nm. In the SCF group, peaks showed steady increase in discriminating power, while in the CF group, the band 1,379 nm showed an increase for 7 days, but then it dropped for 14 days model. There is also a difference in the absorbance bands featured as influential in models for CF in SCF in the area 1,484–1,510 nm. In the case of CF group, there are three different peaks located at 1,484, 1,497, and 1,509 nm, but for SCF group, there is just one broad band located at 1,503 nm.

The interesting aspect of this analysis is that despite different storage conditions, the same absorbance bands were shown as the most important for the description of how strawberries change during cold storage, and yet the analysis also captured the specifics of water molecular reorganization in fruit over time, as a function of different cooling technology.

### Partial least squares regression analysis of water spectral changes during storage

**FIGURE 6:**
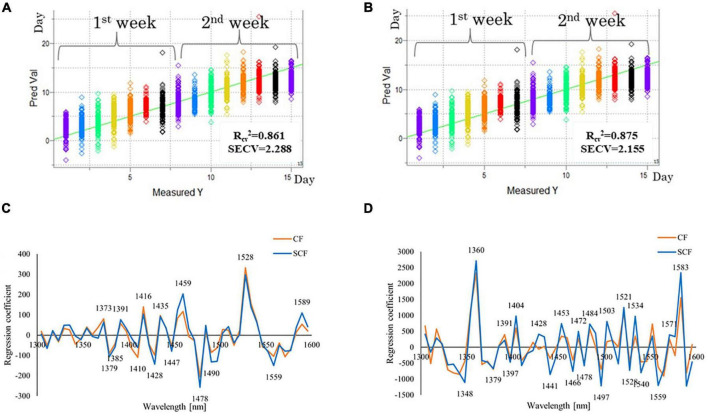
Results of partial least squares (PLS) regression modeling using time as a dependent variable (days of storage). **(A)** Y-fit plot, showing agreement between actual number of storage days and predicted number of storage days using PLS time regression model for spectra of strawberries in the control fridge (CF) condition **(B)** Y-fit plot, showing agreement between actual number of storage days and predicted number of storage days using PLS time regression model for spectra of strawberries in the supercooling fridge (SCF) condition **(C)** regression vectors of PLSR models built using spectral data of strawberry stored in CF and SCF conditions - comparison between regression vectors of models for the first 4 days of storage; **(D)** regression vectors of PLSR models built using spectral data of strawberry stored in CF and SCF conditions - comparison between regression vectors of models for the 15 days of storage.

According to the difference spectra results, which showed different trends of the spectral changes between the first 4 days and later storage period, the PLS regression analysis was first performed using spectral data collected during the first 4 days, and second, using all 14 days spectra [including the spectra collected before placing the strawberries in the storage (day 0)]. The dependent variable in both cases was time, expressed as “days of storage”. The PLSR models were then calculated for CF and SCF data separately, and their performances were compared (Figure 6).

The predictions of the days of storage were successful, regardless of storage conditions with slightly less accuracy for the CF (coefficient of determination *R*_*cv*_^2^ = 0.861, standard error of cross-validation SECV = 2.288) compared to SCF (coefficient of determination *R*_*cv*_^2^ = 0.875, standard error of cross-validation SECV = 2.155 days). The PLSR results showed that the actual day of storage can be predicted with around 2 days error. The Y-fit plots that show agreement between actual and predicted day of storage show a very good linear relationship for both CF (Figure 6A) and SCF (Figure 6B) for all 14 days. The cause of error can be attributed to the differences between the individual samples which can be expected since the models were developed using raw spectral data. The adequate spectral pretreatment aimed at removal of influence of physical differences between the samples (such as surface curvature of the fruit) can probably lead to decreased error.

The results revealed that in the same time span both models for CF and SCF provided similar regression vectors as presented in Figure 6C for 4 days and Figure 6D for 14 days PLSR models. The time regression models for 14 days of storage fit linear pattern, describing the spectral change over the time with very high coefficient of determination (*R*^2^ > 0.85; Figures 6A,B). This is the second analysis that confirms the ability of the NIR spectra to capture the information related to the passage of time in cold storage, and the potential for the estimation of freshness of strawberries.

The regression vectors shown in Figure 6C (for 4 days models) and Figure 6D (for 14 days models) can provide information which variables, i.e., absorbance bands were the most informative for modeling of storage time. As can be seen from these figures, in the first 4 days of the storage (Figure 6C), 1,528 nm appeared as a dominant peak with the highest, positive regression coefficient for both CF and SCF groups. In the model of 14 days of data (Figure 6D), 1,360 and 1,583 nm became much more prominent. There is also significant difference in the overall magnitude of regression vectors coefficients for 4 days models and 14 days models, which are almost eight times larger in the second case. There is also a difference between these models when it comes to the sign of regression vector coefficients – the peaks located at 1,373, 1,391–1,398, 1,416, and 1,528 nm were positive at first, during the 4 days period, but then become negative for the longer storage data. In comparison, the regression coefficient of 1,435 nm stayed positive in all sets, but with less weight.

The differences in regression vectors from the aspect of cooling systems are visible for the 4 days models as well as in 14 days models. The differences appear at 1,350–1,373, 1,391–1,400 nm, especially at 1,459, 1,490–1,500, and 1,559–1,583 nm for the 4 days model (Figure 6C). For the 14 days models, the differences in regression vectors appear at 1,330–1,348, 1,391, especially at 1,428 nm which shows almost no correlation with time for the CF model, and 1,441, 1,453, 1,466, 1,484, and 1,497 nm, again, very large difference at 1,503 and 1,550 nm, and finally again large difference at around 1,580 nm, where even the sign of the regression coefficients for CF and SCF is the opposite.

Taken together, this exploratory analysis of influential variables in PLS regression models show that the spectra of strawberries capture events on a molecular level in the fruit as it is stored, which seems to be common irrespective of the storage conditions. However, they also show distinct differences in effects of cooling systems, at specific absorbance bands which were already observed during previous analyses.

### Choosing water matrix coordinates for aquagram display of water spectral patterns of strawberries

The analysis of spectral data of stored strawberries, despite differences in storage conditions, showed repeating and consistent absorbance bands at which the absorbance changed as a function of storage time. When all the bands from different analyses were put together (Supplementary Table 1), the importance of certain absorbance bands for description of changes in strawberries during storage, based on the number of their appearances as important variables in the analyses became evident.

Out of 28 absorbance bands found important for the description of changes during cold storage, 24 belong to well-known WAMACs, that is, small wavelength ranges 6–20 nm in length that corresponds to the absorbance of specific water molecular conformations (57, 58, 66) (Table 1). Additionally, four absorbance bands featured prominently in the analysis – 1,503, 1,528, 1,534, and 1,559 nm. These bands are not yet recognized as WAMACs. All four bands are located within the part of the NIR spectra where it is known that strongly hydrogen-bonded water absorbs. These four bands carried much weight in the analysis indicating the importance of their distinction and we tentatively labeled them as new WAMACs Ci, Cj, Ck, and Cl that may be of interest to monitor freshness of the agricultural products in general (Table 1).

**TABLE 1:** Water matrix coordinates (WAMACs) that carry information about the changes in strawberry during cold storage [Number of stars “*” in the column Importance represents the number of occurrences of the absorbance band as an influential variable during the whole analysis, as summarized in detail in Supplementary Table 1. Unless otherwise indicated in the table, the source of all information are references (58, 66)].

Absorbance band (nm)	Water matrix coordinates (WAMACs)	Importance	Assignment
1348	**C1** (1,336–1,348 nm)	**	Antisymmetric OH stretch of H_2_O (ν3)
1360	**C2** (1,360–1,366 nm)	***	Water solvation shell OH-(H_2_O)_1,2,4_ Water vapor (59) and proton hydrates (44)
1373	**C3** (1,370–1,376 nm)	***	Combination of symmetric and asymmetric OH stretch of H_2_O (ν1 + ν3) Water vapor (59) and proton hydrates (44)
1379 1385	**C4** (1,380–1,388 nm)	**** ****	Water solvation shell, OH–(H_2_O)_1,4_ and/or superoxide O_2_–(H_2_O)_4_ Water vapor (59) and proton hydrates (44)
1391 and 1397	**C5** (1,396–1,403 nm)	****	Confined single water molecules (trapped water) (76) First overtone of the free OH group trapped in the hydrophobic interior (109)
1404, 1410, and 1416	**C5** (1,404–1,414 nm)	** *** *	Free water molecules
1428	**C6** (1,421–1,430 nm)	***	Water hydration band, H–OH bend hydroxide First overtone of the fundamental OH stretching vibration in water (the water molecules are condensed in one or more layers on sorption sites in the amorphous region) (81)
1435 and 1441	**C7** (1,432–1,444 nm)	* *	Non-bonded O–H stretching first overtone; overtone of the OH bending mode of H_5_O_2_+ water dimer
1447 and 1453	**C8** (1,448–1,454 nm)	***** *	Water solvation shell, OH–(H_2_O)_4,5_
1459 and 1466	**C9** (1,458–1,468 nm)	*** *	Water molecules with two hydrogen bonds
1472 and 1478	**C10** (1,472–1,482 nm)	* ****	Water molecules with three hydrogen bonds
1484, 1490, and 1497	**C11** (1,482–1,495 nm)	** ** **	Water molecules with four hydrogen bonds
1503	**Ci**	****	Strongly bound water, intermolecular hydrogen bonds (42, 110) Hydrogen bonded water molecules participating in the crystal structure (111) OH stretching vibration in Ice III (112)
1509 and 1521	**C12** (1,506–1,516 nm)	* *	Strongly bound water, symmetrical stretching fundamental vibration and doubly degenerate bending fundamental (ν1, ν2)
**1528**	**Cj**	****	Strongly bound water, intermolecular hydrogen bonds (113)
**1534**	**Ck**	*****	Hydrogen bonded hydroxyl groups (–O–H^δ^^+⋅⋅⋅^O^δ^^–^–) (114) First over. of hydrogen bonded O-H stretching (115) Bending fundamental, 2δ_*HOH*_ (103)
**1559**	**Cl**	****	Ionic bound water molecules 1st overt (99, 116) Strongly hydrogen bonded water (117) Crystalline water ice (118)

Judging by the importance of the WAMACs, it can be observed that C4, C5, C8, and C10 and newly defined Ci, Cj, Ck, and Cl (rows highlighted in gray in Table 1) are not only important for the description of the changes during storage but also reflect the differences in strawberries as a function of different cooling storage treatment.

Together, these WAMACs could be thought of multidimensional biomarker that can be used to describe the process of change in strawberries during storage.

### Aquagrams – Water spectral patterns of strawberries during cold storage

**FIGURE 7:**
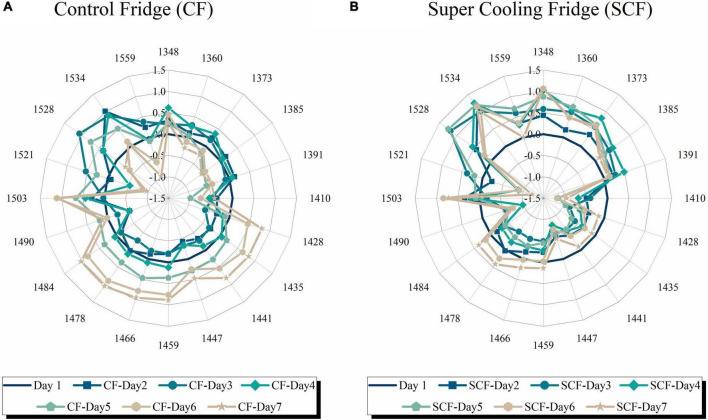
Aquagrams of strawberries showing changes in water molecular matrix of the fruit flesh during 7 days of storage in: **(A)** Control fridge (CF) and **(B)** supercooling fridge.

Based on the previously found WAMACs, 19 representative water absorbance bands were selected to visualize the collective spectral change during storage of strawberries. These absorbance bands define radial axes on aquagrams on which values of difference in normalized absorbance between the respective day and the first day of storage were plotted, as presented in Figure 7, for cold storage in the control fridge (Figure 7A) and supercooling fridge (Figure 7B). For simplicity and practical reasons, the aquagrams are displayed for the 7 days, because of the usual shelf-life of strawberries of 5 days in cold storage.

The comparison of how water spectral pattern of strawberries change during cold storage provided stark differences depending on whether the strawberries were kept in control or supercooling fridge. First, in the area of highly active water species, which are vapor-like (57) absorbing in the region 1,348–1,385 nm, it can be observed that when strawberries are in the CF the absorbance decreases with time, while in the SCF the absorbance is increased and stays relatively stable during this 1 week. In the CF, there is particularly decrease at 1,373 and 1,385 nm on days 5–7. All these vapor-like, weakly bonded water species (water molecules in proton and ion hydration shells, water species involved with hydration of soluble charged compounds) are with high energy and very mobile (56). The observed difference suggests increased content of soluble compounds in strawberries stored in SCF, active respiration, and exchange of water in the gaseous phase with the environment and, since the bands 1,360, 1,373, and 1,385 nm are the signature bands of moisture and water activity (56), it also implies the existence of water available to participate in necessary biochemical reactions.

The differences are also present at the bands of free (1,410 nm) and quazi-free water (1,391 nm). For the case of CF, after the initial period of no changes at 1,391 nm, absorbance decreases after fifth day in storage. On the contrary, the absorbance at this band is increased for the case of SCF and declines gradually. This absorbance band is related to trapped water, either in between hydration shells of ions or in the hydrophobic interior, which implies that there is the ongoing production of some compounds in strawberries during this time, which results in their hydration and subsequently confined interior in which single water molecules get confined. This would be in agreement with increased absorbance at hydration shells bands (1,360 and 1,385 nm) signaling the increase in soluble compounds observed previously. Taken together, this implies that upon cold storage in SCF, the strawberries show more signs of active metabolism and production of soluble compounds. It is interesting to point out that the close absorbance band located at 1,398 nm, which is also attributed to trapped water, was found to be highly correlated with vitamin C content and shrinkage of fruit during hot air drying (77).

The absorbance of free water molecules (1,410 nm) is decreased immediately upon storage in both fridges; however, there is difference in the magnitude of this decrease. The decrease is much stronger, very sharp and regular in the case of SCF, while it is not regular and shows fluctuations in CF. This is most probably heavily related to the observed weight loss of strawberries and can be indicated loss of juiciness (56). It was previously observed that decrease in free water molecules is an important defense reaction in response to desiccation stress and that decrease of free water molecules might be the survival strategy in plants in the stress conditions (78). Since a decrease of free molecules is the common reaction in strawberry for both cold storage conditions, this might indeed be the case; however, the gradual and regular decrease in the case of SCF indicates organized control of water loss [also a part of survival strategy as found before (78)].

The largest differences in aquagrams could be observed in the area from 1,428 to 1,490 nm. In the SCF, the absorbance of strawberries seemed to be decreased immediately upon storage and kept that way, and only on days 6 and 7, there is increase at the bands from 1,459 to 1,484 nm. However, such decrease of absorbance was not observed for the CF, and in contrast, the absorbance shows huge increase at all these bands starting from day 4. The band at 1,428 nm, ascribed to hydration water, belongs to the WAMACS C6. In numerous studies, this band was attributed to amorphous regions in cellulose (79–84); however, in the case of strawberry, this water is associated with other polysaccharides. It seems that absorbance increase at this band is associated with drying and increased density (80, 85). There are several research studies that report bands from this particular WAMACS region, as important for the changes during processing and storage. For example, band 1,420 nm was found to be the optimal wavelength for vitamin C content prediction in apple slices during drying (77) and also had high correlation with internal quality of fruit (86, 87). Absorbance at 1,420 nm was in another study reported to be closely related to weight loss during the storage of mushrooms (88), while in another study, it was found that the absorbance at this band particularly increases as a function of storage time [similar to the change of soluble solids content (SSC)] (87). It is worth noting that, in difference spectra, there was actually a minimal change in absorbance at 1,416 nm in the case of CF and 1,422 nm in the case of SCF. While it is not clear what exactly is the difference between the bands 1,416, 1,422, and 1,428 nm, seeing that they are usually attributed to the same water molecular conformation, it is worth noting that this WAMACS region is repeatedly showing the importance of the changes during storage and internal quality of vegetable and fruits. The stark difference in behavior of absorbance at the band 1,428 nm, depending on whether strawberries were stored in CF and SCF seems to further indicate the importance of this band for preservation in cold storage, most probably because it is related to the state of turgor and cytoplasm in the cells, its shrinkage and pulling away from the cell walls. That may explain why the bands from this WAMACS region were so often found related to internal quality, damage, and vitamin C production, seeing that production of vitamin C is the part of the protection against damage. The behavior of absorbance at this band in the case of CF seems to be in agreement with the typical changes during storage; however, the opposite behavior in the case of SCF seems to indicate the presence of electric field slows down this process.

The absorbance bands from 1,435 to 1,490 nm can be related to the water molecular species interacting with biomolecules (1,435 nm), water species participating in hydration (1,459 nm) and water molecules bonding to each other, i.e., creating clusters with 1–4 hydrogen bonds. In the research about the texture of apples, it was found that water spectral pattern characterized by the low absorbance in the region 1,344–1,382 nm and high at 1,410–1,492 nm corresponds to the firm, juicy, and crispy sensory profile (36). The difference in these two particular absorbance regions, in the case of strawberries, might indicate that the presence of electric field actually slows down the process of post-harvest ripening of strawberries in the cold storage.

Next, common for both refrigeration conditions, there is a sharp decrease in absorbance at 1,492 nm, water absorbance band of water molecules with four hydrogen bonds, one of the two bands, the other one being 1,410 nm, free water molecules, related to the influence of temperature (89). For pure water, if the temperature increases the absorbance at the band 1,492 nm will decrease, while at 1,410 nm increase, because the temperature causes breaking of the hydrogen bonds in tetrahedral structure creating more free molecules. It is, therefore, interesting that in strawberry after the fruit was placed in the cold storage there was a decrease in absorbance at this particular band. This might indicate that there is an innate mechanism in the fruit which fights the creation of this type of hydrogen-bonded water. However, it seems that with the progress of time, this mechanism fails in the case of the CF, where increase in absorbance can be observed after day 4, which suggests creation of seeds of ice crystals that may become the source of damage. From this aspect, the presence of electric field in the SCF seems to act protectively.

The behavior of absorbance at band 1,503 nm is common for both storage conditions; there is a sharp, strong increase in absorbance immediately upon the storage. There are many reports about this particular band in characterization of minerals and attributed to vibrational mode of hydrous defects in the crystal structure (90–96). In analogy to these reports, the absorbance at this band can be considered as indicator of shallow, superficial, surface damage, and small openings in the structure of materials that enhance exposure to the environment. If this is correct interpretation that would mean some surface damage on the strawberry skin appeared in both storage conditions, leaving the fruit vulnerable. The region beyond 1,503 nm, encompassing bands from 1,521 to 1,559 nm includes absorbance bands of water species that are tightly bound to the structural elements of the fruit tissue, including crystalline and polymer-bound water (97–100) and represent the water that cannot be lost to processes such as drying or squeezing (101); this water is lost only if the structural elements associated with it are lost. The difference between the water species absorbing at particular bands is in the level of complexity of the structure they are engaged in, and in the strength of the bonds, which is increased with the increase in wavelength. Having this in mind, it seems that in both cold storage conditions, the strawberries suffered some level of tissue damage, seeing a sharp decrease at 1,521 and 1,559 nm bands. The band at 1,534 nm was often attributed to strongly bound water (102, 103) related to water–polymers interaction [such as cellulose (104)] that appears during initial phases of drying (105), while the band at 1,559 nm is typically assigned to bond vibration of O–H stretch in crystalline cellulose (104, 106). Here, it is important to note that while other reports are attributing these bands to cellulose, they are in fact water absorbance bands, and in the case of strawberries, this strongly bound water is bound to numerous polysaccharides, typical building block of cell walls. With the progress of time in storage, the decrease in absorbance can be observed at all those bands in the case of CF, but not in the case of SCF.

The second most striking difference between the spectral patterns of strawberries in CF and SCF seems to be exactly the decrease in absorbance at the bands of strongly bound water. This seems to be connected with the increase in absorbance of the so-called bulk water, all those small clusters of hydrogen-bonded water species observed earlier in the region 1,435–1,484 nm, which happens in the case of the CF. While the strongly bound water, as the term itself clearly indicates, is strongly bound to structures in the fruit, the bulk water is water not associated to fruit, it is the “water hydrating other water molecules”. The decrease in strongly bound water can be connected to softening of the fruit during ripening and increased bulk water with swelling and expansion of vacuolar volume (107). Since these changes are not present in the SCF, it suggests that ripening of strawberries is delayed by the presence of electric field.

**FIGURE 8:**
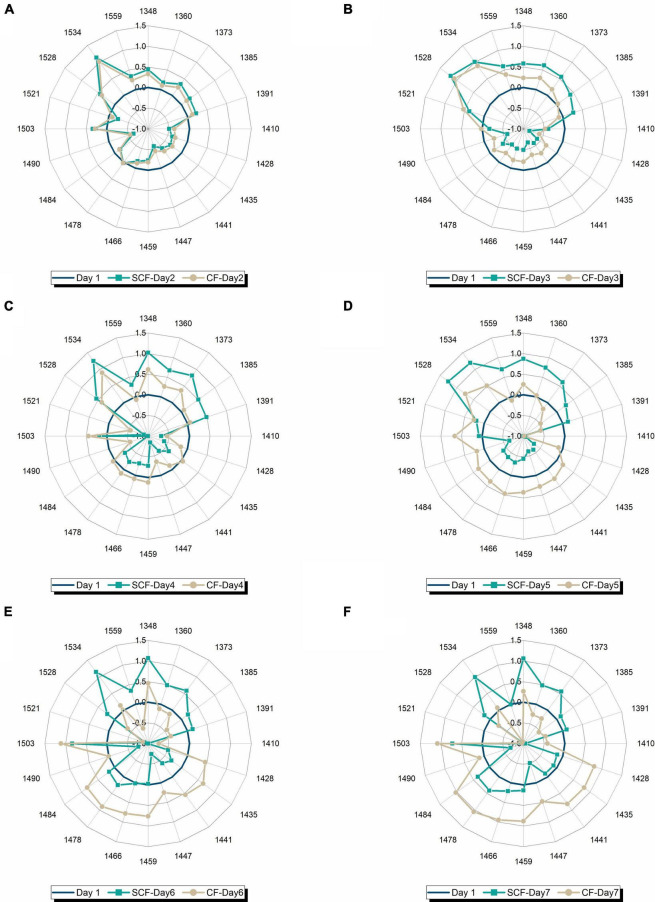
Comparison of water spectral patterns of strawberries stored in control (CF) and supercooling fridge (SCF) on day-to-day basis: **(A)** After 1 day of storage, **(B)** after 2 days of storage, **(C)** after 3 days of storage, **(D)** after 4 days of storage, **(E)** after 5 days of storage, and **(F)** after 1 week of cold storage.

**FIGURE 9:**
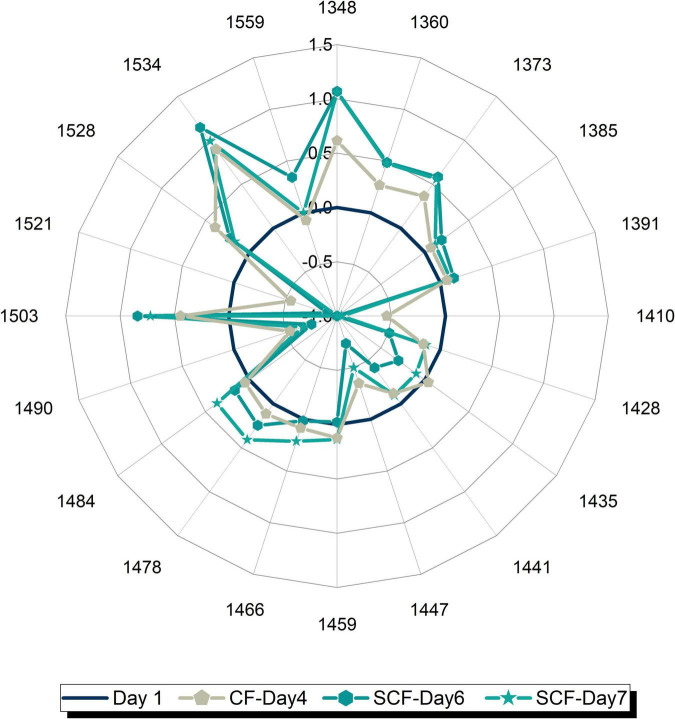
Comparison of water spectral patterns of strawberries in the control fridge (CF) on day 4 and strawberries in supercooling fridge (SCF) on days 6 and 7 shows that the presence of electric field delays the ripening in cold storage.

To examine better this possibility and see better the transformation of water spectral pattern over time, a series of aquagrams was created showing comparison of water spectral patterns of strawberries in CF and SCF, 1 day at a time (Figures 8A–F). From this series of plots, the first interesting observation is that the patterns after 1 day of storage (Figure 8A) are nearly identical, testifying to the equality of the samples at the beginning of the experimental period, while the last plot (Figure 8F) shows remarkably well how different storage conditions led to the completely different water spectral patterns, i.e., how spectral pattern as a multidimensional marker accurately depicted the difference in the final state of the strawberries caused by different storage conditions. The first changes can be observed on day 3 of storage (Figure 8B) where higher absorbance can be observed at strongly bound water bands (1,528–1,559 nm) and at vapor-like and trapped water (1,348–1,391 nm) in the case of strawberries in SCF. This increase is continued over subsequent days (Figures 8C–F), while the spectral pattern of strawberries in CF is primarily characterized by decrease of absorbance at strongly bound water bands, and large increase in absorbance of bulk water. But the patterns become more and more similar, for example, the pattern of strawberries in SCF fridge on day 7 (Figure 8F) looks very much like the pattern of strawberries in CF fridge on day 4 (Figure 8C). This strongly suggests the fruit is going through the similar process, but with some time delay in the case of SCF fridge. To test this assumption, one more aquagram is created (Figure 9), where the water spectral patterns of strawberries on days 6 and 7 in SCF are compared to the spectral pattern of strawberries in CF on day 4. The spectral patterns show strikingly similar shape, indicating that the fruit indeed goes through a similar process, which is in SCF delayed for around 3 days. The possible mechanism of how this happens might be explained by the influence of electric field on the surface hardening of the strawberries, which limit the water diffusion and thus evaporation, as research findings reported to be the case in electric field treated apples post-harvest (108). This would be very consistent with the observed increased absorbance of strongly bound water and conserved vapor-like water within strawberries in SCF.

The important finding in this study also is that the water spectral pattern as a multidimensional biomarker captures perfectly the process of ripening and shows that it can be described only by using water absorbance bands. The consistently found importance of these particular bands was confirmed through several analyses and they can be considered as WAMACS or water matrix coordinates that define the space of variables describing the changes of postharvest ripening that can probably be used for other biological systems as well. Numerous research works have already found these bands related to certain functionalities in other bio-aqueous systems, and this information is summarized in Table 2.

**TABLE 2:** Water matrix coordinates (WAMACS) that can be used to describe the changes during post-harvest ripening of strawberries during cold storage.

Absorbance band (nm)	Roles/Functionality/Importance in certain biological processes
1348	Self-organization (44), germination (42), changes during storage (56), and viability (119)
1360	Germination (42), water activity and changes during storage (56), hardness (120), viability (119), and texture (36)
1373	One of the bands of water vapor, proton hydration/indicator of changes in sucrose content (121), viability (119), water activity and changes during storage (56), and texture (36)
1385	Hydration of ions/important for pH modeling (122), and firmness and texture (36)
1391	Drying, dehydration, expulsion of cellular water, damage, stress, infection (101, 119, 123); and indicate the amount of vitamin C content (124)
1410	Moisture content, water activity, seed viability, firmness (125, 126); and indicate the amount of vitamin C content (124)
1428	Protein hydration, protein folding (127), water activity (56, 128), damage, and defects
1435	Phase transition, carbohydrates–water interaction, hardness, and viability (56)
1441	Preservation during desiccation stress (78)
1447	Water activity, biotic and abiotic stress indicator, preservation, and damage (36, 37, 40–42, 56)
1459	Water activity, biotic and abiotic stress indicator, preservation, and damage (36, 37, 40–42, 56)
1466	Protein–water interaction (127, 129)
1478	Semi-crystalline water associated with cellulose (81) or other polymers related to mechanical properties like stiffness (81)
1484	Associated with cellulose (104, 130, 131) or other polymers indicator of preservation of tissues, damage (113)
1490	Water activity, biotic and abiotic stress indicator, preservation, and damage (36, 37, 40–42, 56)
1503	Skin disease in plants and animals (132, 133) defects in minerals (90–96, 134), and structural defects in starch (135)
1521	Structural water; water–cellulose interaction (104), water–carbohydrate interaction (136), water–fibre (starch) interaction (137), important for discrimination of fresh/thawed meat, characteristic of fresh meat (138, 139), associated with differences in level of mechanical damage (113)
1528	Structural water; water–cellulose interaction (104), water–carbohydrate interaction (136), water–fiber (starch) interaction (137), important for discrimination of fresh/thawed meat, characteristic of fresh meat (138, 139), associated with differences in level of mechanical damage (113)
1534	Structural water; water–cellulose interaction (104), indicator of drying (105), water–protein interaction, water sorption, and clustering of water sorbed molecules (103)
1559	Structural water; associated with starch or cellulose (140, 141) and other polymers (106, 142, 143), associated with sugars (142, 144–146), associated with proteins (147) or amino compounds (148), associated with membrane structure, influenced by temperature (117), related to mechanical properties (106), indicator of fermentation (149), indicative of vegetative growth stage (142), indicator of crystallinity (118, 150), indicator of preservation of tissues, damage (113)

Currently available information and the tentative functionality of corresponding water species in biological and aqueous systems.

## Conclusion

This study aimed at investigating the spectral changes during the storage of strawberry in order to confirm the potential of aquaphotomics NIRS for monitoring during cold storage. Further, the same technology was applied to assess the influence of the storage environments with a particular objective to find and explain the differences in applied cooling systems [without (CF) and with the electric field exposure (SCF)] on the stored fruit. The water matrix transformation during the post-harvest degradation of strawberry as well as the influence of electric field could be observed using the NIR spectra of strawberries in the 1,300–1,600 nm region.

Over the course of several analyses, the same water absorbance bands were found to be important for the description of the changes in fruit during storage. The main outcomes of the analyses were that it was possible to detect spectral changes over storage and predict the time spent in storage. The consistently repeating water absorbance bands were used to define water spectral patterns as biomarkers of the state of the strawberries in cold storage and allowed comparison of strawberries stored in different conditions. This comparison showed that the presence of electric field in supercooling fridge slows down the ripening processing, delaying it for about 3 days. The possible mechanism behind can be explained by the influence of electric field on skin hardening, which then acts as a natural coating and slows down evaporation. This agreed with the lower water loss percentage recorded for strawberries in the supercooling fridge.

Using this technique of spectral monitoring, the ripening process can be observed in a non-destructive way. This provides the potential of aquaphotomics using NIR for the post-harvest monitoring, and quality evaluation of strawberry and other agricultural products at different storage conditions.

## Data availability statement

The raw data supporting the conclusions of this article will be made available by the authors, without undue reservation.

## Author contributions

TF, TK, and RT: conceptualization and resources. RT, SA, and JM: methodology and investigation. SA: software. SA, TF, and RT: validation. SA and JM: formal analysis, writing—original draft preparation, and visualization. SA and RT: data curation. JM: writing—review and editing. RT: supervision and project administration. RT and TK: funding acquisition. All authors read and agreed to the published version of the manuscript.
