# Supplementary material for: Aquaphotomics monitoring of strawberry fruit during cold storage – A comparison of two cooling systems
Source: Front Nutr. 2022 Dec 9;9:1058173. doi: 10.3389/fnut.2022.1058173 (PMC9780392; doi:10.3389/fnut.2022.1058173)
Supplement: Supplementary file 1 [file Table_1.docx]

Supplementary Material

# Supplementary Data

Table S1. The absorbance bands that showed importance for storage monitoring and/or discrimination between the type of cold storage of strawberries, and their positions within the previously discovered water matrix coordinate (WAMACS) ranges (1), with tentative proposal for 4 new WAMACS

| Absorbance band (nm) | Source (analysis) | WAMACS (1–3) |
| --- | --- | --- |
| 1311 | SIMCA results for CF group (Fig.3a) |  |
| 1311 | SIMCA results for SCF group (Fig.3b) |  |
| 1323 | SIMCA results for CF group (Fig.3a) |  |
| 1329 | PLSR model for 15 days storage period (Fig.4d) |  |
| **1348** | The difference spectra of SCF during the storage (Fig.2d) | **C1** (1336-1348 nm) |
| **1348** | PLSR model for 15 days storage period (Fig.4d) |  |
| 1350 | PLSR model for 4 days storage period (Fig.4c) |  |
| **1360** | SIMCA results for CF group (Fig.3a) | **C2** (1360-1366 nm) |
| **1360** | SIMCA results for SCF group (Fig.3b) |  |
| **1360** | PLSR model for 15 days storage period (Fig.4d) |  |
| **1373** | The difference spectra of CF during the storage (Fig.2c) | **C3** (1370-1376 nm) |
| **1373** | The difference spectra of SCF during the storage (Fig.2d) |  |
| **1373** | PLSR model for 4 days storage period (Fig.4c) |  |
| **1379** | SIMCA results for CF group (Fig.3a) | **C4** (1380-1388 nm) |
| **1379** | SIMCA results for SCF group (Fig. 3b) |  |
| **1379** | PLSR model for 4 days storage period (Fig.4c) |  |
| **1379** | PLSR model for 15 days storage period (Fig.4d) |  |
| **1385** | The difference spectra before storage and after 1 day of storage (Fig.2a) |  |
| **1385** | The difference spectra before and after 4 days of storage (Fig.2b) |  |
| **1385** | The difference spectra of CF during the storage (Fig.2c) |  |
| **1385** | PLSR model for 4 days storage period (Fig.4c) |  |
| **1391** | The difference spectra of SCF during the storage (Fig.2d) | **C5** (1396-1403 nm) |
| **1391** | PLSR model for 4 days storage period (Fig.4c) |  |
| **1391** | PLSR model for 15 days storage period (Fig.4d) |  |
| **1397** | PLSR model for 15 days storage period (Fig.4d) |  |
| **1404** | The difference spectra before storage and after 1 day of storage (Fig.2a) | **C5** (1404-1414 nm) |
| **1404** | PLSR model for 15 days storage period (Fig.4d) |  |
| **1410** | The difference spectra before and after 4 days of storage (Fig.2b) |  |
| **1410** | The difference spectra of CF during the storage (Fig.2c) |  |
| **1410** | PLSR model for 4 days storage period (Fig.4c) |  |
| **1416** | PLSR model for 4 days storage period (Fig.4c) |  |
| **1428** | The difference spectra before and after 4 days of storage (Fig.2b) | **C6** (1421-1430 nm) |
| **1428** | PLSR model for 4 days storage period (Fig.4c) |  |
| **1428** | PLSR model for 15 days storage period (Fig.4d) |  |
| **1435** | PLSR model for 4 days storage period (Fig.4c) | **C7** (1432-1444 nm) |
| **1441** | PLSR model for 15 days storage period (Fig.4d) |  |
| **1447** | The difference spectra before and after 4 days of storage (Fig.2b) | **C8** (1448-1454 nm) |
| **1447** | The difference spectra of SCF during the storage (Fig.2d) |  |
| **1447** | SIMCA results for CF group (Fig. 3a) |  |
| **1447** | SIMCA results for SCF group (Fig. 3b) |  |
| **1447** | PLSR model for 4 days storage period (Fig.4c) |  |
| **1453** | SIMCA results for CF group (Fig. 3a) |  |
| **1453** | PLSR model for 15 days storage period (Fig.4d) |  |
| **1459** | The difference spectra of CF during the storage (Fig.2c) | **C9** (1458-1468 nm) |
| **1459** | The difference spectra of SCF during the storage (Fig.2d) |  |
| **1459** | PLSR model for 4 days storage period (Fig.4c) |  |
| **1466** | PLSR model for 15 days storage period (Fig.4d) |  |
| **1472** | PLSR model for 15 days storage period (Fig.4d) | **C10** (1472-1482 nm) |
| **1478** | The difference spectra of CF during the storage (Fig.2c) |  |
| **1478** | The difference spectra of SCF during the storage (Fig.2d) |  |
| **1478** | PLSR model for 4 days storage period (Fig.4c) |  |
| **1478** | PLSR model for 15 days storage period (Fig.4d) |  |
| **1484** | SIMCA results for CF group (Fig.3a) | **C11** (1482-1495 nm) |
| **1484** | PLSR model for 15 days storage period (Fig.4d) |  |
| **1490** | The difference spectra of SCF during the storage (Fig.2d) |  |
| **1490** | PLSR model for 4 days storage period (Fig.4c) |  |
| **1497** | SIMCA results for CF group (Fig.3a) |  |
| **1497** | PLSR model for 15 days storage period (Fig.4d) |  |
| **1503** | The difference spectra of CF during the storage (Fig.2c) | **Ci** |
| **1503** | The difference spectra of SCF during the storage (Fig.2d) |  |
| **1503** | SIMCA results for SCF group (Fig. 3b) |  |
| **1503** | PLSR model for 15 days storage period (Fig.4d) |  |
| 1509 | SIMCA results for CF group (Fig. 3a) | **C12** (1506-1516 nm) |
| 1521 | PLSR model for 15 days storage period (Fig.4d) |  |
| **1528** | SIMCA results for CF group (Fig.3a) | **Cj** |
| **1528** | SIMCA results for SCF group (Fig.3b) |  |
| **1528** | PLSR model for 4 days storage period (Fig.4c) |  |
| **1528** | PLSR model for 15 days storage period (Fig.4d) |  |
| **1534** | The difference spectra of CF during the storage (Fig.2c) | **Ck** |
| **1534** | The difference spectra of SCF during the storage (Fig.2d) |  |
| **1534** | SIMCA results for CF group (Fig.3a) |  |
| **1534** | SIMCA results for SCF group (Fig.3b) |  |
| **1534** | PLSR model for 15 days storage period (Fig.4d) |  |
| 1540 | PLSR model for 15 days storage period (Fig.4d) |  |
| 1550 | PLSR model for 15 days storage period (Fig.4d) |  |
| **1559** | SIMCA results for CF group (Fig.3a) | **Cl** |
| **1559** | SIMCA results for SCF group (Fig. 3b) |  |
| **1559** | PLSR model for 4 days storage period (Fig.4c) |  |
| **1559** | PLSR model for 15 days storage period (Fig.4d) |  |
| 1571 | PLSR model for 15 days storage period (Fig.4d) |  |
| 1577 | PLSR model for 15 days storage period (Fig.4d) |  |
| 1583 | PLSR model for 15 days storage period (Fig.4d) |  |

Table S2. Tentative proposal of four new WAMACS with their assignment and information about the related functionality in aqueous and biological systems

| Absorbance band (nm) | Potential assignment / Functionality |
| --- | --- |
| 1503 | 1503 nm – strongly bound water, influential variable in modeling of ascorbic acid content in mung bean (in living matrixes, AA has tendency to create efficient inter-molecular hydrogen bonds)(4)  1503 nm – influential variable in prediction of water content in human nails (5)  1503 nm – important variable for early diagnosis of cassava frog skin disease in powdered tissue samples (6)  1503 nm – water absorption wavelength, reflects moisture content in maize leaves (7)  1503 nm - important variable for detection of common scab skin disease of the potato tubers (8)  1503 nm (3326 cm^-1^) – strong intermolecular hydrogen bond in phenols (9)  1503 nm (3326 cm^-1^) – hydrogen bonded, NH stretching vibration in aqueous vinyl polymer solution (10)  1503 nm (3326 cm^-1^) - hydrogen-bonded N–H stretching mode (11)  1503 nm (3326 cm^-1^) – OH stretching vibrations of hydrogen bonded water molecules participating in the crystal structure (12)  1503 nm (3326 cm^-1^) – water stretching vibrations in minerals, in connection with defects (13–15)  1503 nm (3326 cm^-1^) – OH stretching vibration in Ice III (16)  1503 nm (3326 cm^-1^) – H-related defects gives rise to O-H local vibrational mode absorption at 3326 and 3611 cm^-1^ (17)  1503 nm (3326 cm^−1^) - attributed to OH group located in a vacant site; vibrational mode of hydrous defect in wadsleyite mineral; related to incorporation of hydrogen (protonation) (18)  1503 nm (3326 cm^−1^) – hydroxyl group in raw starch material, affected by glutaraldehyde vapor phase crosslinking; becomes wider and weaker (19)  1503 nm (3326 cm^−1^) – OH stretching line assigned to a shallow donor that is introduced by H into ZnO crystals(20)  1503 nm (3326 cm^−1^) – hydrogen defect in ZnO (21)  1503 (3326 cm^−1^ ) – hydrogen bond on O site, in relation to protonation of O on anomalous oxygen site (22) |
| 1528 | 1528 nm – starch OH stretch, hydrogen bonded intramolecular or intermolecular with water molecules; associated with differences in level of mechanical damage (23) |
| 1534 | 1534 nm – one of 3 wavelengths used in multiple linear regression for predicting bread loaf volume (1506, 1534 and 1618 nm); measurement of some parameter related to volume independent of protein (24)  1534 nm – 1^st^ overtone of NH stretching (25)  1534 (3259 cm^-1^) – N-H stretching (26,27)  1534 nm – one of 4 wavelengths (662, 686, 1534 and 1753 nm) used for improved prediction of chlorophyll content in intact canola (28)  1534 nm (3259 cm^-1^) – hydrogen bonded hydroxyl groups (–O–H^δ+^···O^δ−^–) (29)  1534 nm (3259 cm^-1^) the H–O stretching vibrations of the absorbent water (30)  1534 nm (6518 cm^-1^) – 1^st^ over. of hydrogen bonded O-H stretching (31)  1534 (3259 cm^-1^) – one of the 3 water stretching bands observed in carbonate mineral huanghoite by Raman spectroscopy (the other two being 1435 nm (3484 cm^-1^) and 1393 nm (3589 cm^-1^)) (13)  1534 (3259 cm^-1^) – sesquihydrate crystallite (32) (hydrate whose solid contains 3 molecules of water of crystallization per two molecules)  1534 (3259 cm^-1^) – one of the vibrational frequencies that can be assigned to water heptamer (H_2_O)_7_ single H-donor OH stretch (33) |
| 1559 | 1560 nm – first overtone of glucose (34)  1560 nm – glucose band (35)  1559 nm (3207 cm^-1^)-OH group in D-(+)galactose (36)  1560 nm (6410 cm^−1^) - first overtone of the NH bond (calibration model for concentration of amino acid theanine in tea, indicator of fermentation) (37)  1559 nm – protein band in model for estimating soybean meal content in compound feeds (38)  1560 nm (6410 cm^-1^) -Amine b, Overtone of primary NH_2_ plus secondary NH stretching (39)  1560–1570 nm region- associations with starch and sugar, due to stretching O–H bonds, bands important for classification of vegetative growth stage in snapbean (40)  1560 nm – hydrogen bonded water, influenced by temperature in model membranes (41)  1560 nm (6410 cm^-1^) crystalline water ice feature (42)  1560 nm (6410 cm^-1^) – intensity decrease is an indicator of crystallinity decrease for a model polymorphic drug (43)  1560 nm (3205 cm-1)- strongly hydrogen-bonded water species (44)  1560 nm (3205 cm-1)-hydrogen bonded water to the chains of super absorbent polymer network chains (dewatering coal) (45)  1560 nm (3205 cm^-1^)-vibrations of the hydrogen-bonded water OH groups forming the ring structure (in naphtol-water clusters 1-naphtol·(H_2_O)_3_) (46)  1560 nm (3205 cm^-1^)-stretching of adsorbed water in pollen grains increasing with increased relative humidity (47)  1560 nm (3205 cm^-1^)-H-bonded O-H stretch (48,49)  1560 nm (3205 cm^-1^)- water pentamer (50)  1559 nm (3207 cm^-1^)-intramolecular hydrogen bonding from -OH···N or from OH···O (51)  1560 nm (3205 cm^-1^)-O-H asymmetric stretch in Zundel cation H_5_O_2_+(52)  1560 nm (3205 cm^-1^)-cyclic multimer (53)  1560 nm (3205 cm^-1^)-OH mode of strongly bounded adsorbed water molecules in cellulose (54,55)  1560 nm (3205 cm^-1^)- strongly hydrogen bonded water, water coordinated to cations (56) |

# References

1. Tsenkova R. Aquaphotomics: Dynamic spectroscopy of aqueous and biological systems describes peculiarities of water. *J Near Infrared Spectrosc* (2009) 17:303–313. doi: 10.1255/jnirs.869

2. van de Kraats EB, Munćan J, Tsenkova RN. Aquaphotomics – Origin, concept, applications and future perspectives. *Substantia* (2019)13–28. doi: 10.13128/substantia-702

3. Muncan J, Tsenkova R. Aquaphotomics-From Innovative Knowledge to Integrative Platform in Science and Technology. *Molecules* (2019) 24:2742. doi: 10.3390/molecules24152742

4. Nugraha DT, Zaukuu JLZ, Bósquez JPA, Bodor Z, Vitalis F, Kovacs Z. Near-Infrared Spectroscopy and Aquaphotomics for Monitoring Mung Bean (Vigna radiata) Sprout Growth and Validation of Ascorbic Acid Content. *Sensors 2021, Vol 21, Page 611* (2021) 21:611. doi: 10.3390/S21020611

5. Egawa M, Fukuhara T, Takahashi M, Ozaki Y. Determining water content in human nails with a portable near-infrared spectrometer. *Appl Spectrosc* (2003) 57:473–478. doi: 10.1366/00037020360626032

6. Freitas EL, Brito AC, de Oliveira SAS, de Oliveira EJ. Early diagnosis of cassava frog skin disease in powdered tissue samples using near-infrared spectroscopy. *Eur J Plant Pathol* (2020) 156:547–558. doi: 10.1007/S10658-019-01904-X/FIGURES/4

7. Xiang C, Minzan L, Hong S, Wei Y, Junyi Z, Bohui M. Rapid determination of moisture content in maize leaf based on transmission spectrum. *Trans Chinese Soc Agric Eng* (2017) 33:137–142.

8. Dacal-Nieto A, Formella A, Carrión P, Vazquez-Fernandez E, Fernández-Delgado M. “Common scab detection on potatoes using an infrared hyperspectral imaging system.,” In: Maino G, Foresti GL, editors. *Image Analysis and Processing – ICIAP 2011. ICIAP 2011. Lecture Notes in Computer Science*. Berlin, Heidelberg: Springer (2011). p. 303–312 doi: 10.1007/978-3-642-24088-1_32

9. Solcaniova E, Kovac S. Hydrogen Bonding in Phenols . IV . Intramolecular OH .. . n Hydrogen Bonds of Some Alkyl Derivatives. *Chem Zvesti* (1969) 691:687–691.

10. Ling Z, Omura Y, Hori N, Iwata T, Takemura A. In-situ chemical structure analysis of aqueous vinyl polymer solution-isocyanate adhesive in post-cure process by using Fourier transform near infrared spectroscopy. *Int J Adhes Adhes* (2018) 81:56–64. doi: 10.1016/J.IJADHADH.2017.11.007

11. Tang CY, Kwon YN, Leckie JO. Probing the nano- and micro-scales of reverse osmosis membranes—A comprehensive characterization of physiochemical properties of uncoated and coated membranes by XPS, TEM, ATR-FTIR, and streaming potential measurements. *J Memb Sci* (2007) 287:146–156. doi: 10.1016/J.MEMSCI.2006.10.038

12. Frost RL, Dickfos MJ, Čejka J. Raman spectroscopic study of the uranyl carbonate mineral zellerite. *J Raman Spectrosc* (2008) 39:582–586. doi: 10.1002/JRS.1879

13. Frost RL, Scholz R, Lõpez A. Raman and infrared spectroscopic characterization of the arsenate-bearing mineral tangdanite– and in comparison with the discredited mineral clinotyrolite. *J Raman Spectrosc* (2015) 46:920–926. doi: 10.1002/JRS.4691

14. Rémazeilles C, Refait P. Fe(II) hydroxycarbonate Fe2(OH)2CO3 (chukanovite) as iron corrosion product: Synthesis and study by Fourier Transform Infrared Spectroscopy. *Polyhedron* (2009) 28:749–756. doi: 10.1016/J.POLY.2008.12.034

15. Litasov K, Ohtani E. Systematic Study Of Hydrogen Incorporation Into Fe‐bearing Wadsleyite And Water Storage Capacity Of The Transition Zone. *AIP Conf Proc* (2008) 987:113. doi: 10.1063/1.2896954

16. Bertie JE, Whalley E. Infrared spectra of ices II, III, and V in the range 4000 to 350 cm -1. *J Chem Phys* (1964) 40:1646–1659. doi: 10.1063/1.1725374

17. Shi GA, Stavola M, Pearton SJ, Thieme M, Lavrov E V., Weber J. Hydrogen local modes and shallow donors in ZnO. *Phys Rev B - Condens Matter Mater Phys* (2005) 72:195211. doi: 10.1103/PHYSREVB.72.195211/FIGURES/6/MEDIUM

18. Walker AM, Demouchy S, Wright K. Computer modelling of the energies and vibrational properties of hydroxyl groups in α- and β-Mg2SiO4. *Eur J Mineral* (2006) 18:529–543. doi: 10.1127/0935-1221/2006/0018-0529

19. Wang W, Jin X, Zhu Y, Zhu C, Yang J, Wang H, Lin T. Effect of vapor-phase glutaraldehyde crosslinking on electrospun starch fibers. *Carbohydr Polym* (2016) 140:356–361. doi: 10.1016/J.CARBPOL.2015.12.061

20. Shi GA, Saboktakin M, Stavola M, Pearton SJ. “Hidden hydrogen” in as-grown ZnO. *Appl Phys Lett* (2004) 85:5601. doi: 10.1063/1.1832736

21. Herklotz F, Chaplygin I, Lavrov E V., Neiman A, Reeves RJ, Allen MW. Bistability of a hydrogen defect with a vibrational mode at 3326cm-1 in ZnO. *Phys Rev B* (2019) 99:115203. doi: 10.1103/PHYSREVB.99.115203/FIGURES/7/MEDIUM

22. Jacobsen SD, Demouchy S, Frost DJ, Ballaran TB, Kung J. A systematic study of OH in hydrous wadsleyite from polarized FTIR spectroscopy and single-crystal X-ray diffraction: Oxygen sites for hydrogen storage in Earth’s interior. *Am Mineral* (2005) 90:61–70. doi: 10.2138/AM.2005.1624

23. Osborne BG. Near infrared spectroscopic studies of starch and water in some processed cereal foods. *J Near Infrared Spectrosc* (1996) 4:195–200. doi: 10.1255/jnirs.90

24. Rubenthaler GL, Pomeranz Y. Near-Infrared reflectance spectra of hard red winter wheats varying widely in protein content and breadmaking potential. *Cereal Chem* (1987) 64:407–411.

25. Tran CD, Gao GH. Determination of Monomethylhydrazine with a High-Throughput, All-Fiber Near-Infrared Spectrometer Based on an Integrated Acoustooptic Tunable Filter and an Erbium-Doped Fiber Amplifier. *Anal Chem* (1997) 69:1461–1464. doi: 10.1021/AC960919U/ASSET/IMAGES/MEDIUM/AC960919UE00001.GIF

26. Ibitoye EB, Lokman IH, Hezmee MNM, Goh YM, Zuki ABZ, Jimoh AA. Extraction and physicochemical characterization of chitin and chitosan isolated from house cricket. *Biomed Mater* (2018) 13:025009. doi: 10.1088/1748-605X/AA9DDE

27. Ma S, Wang M, Liu Y, Yang C, Chi L, Xu Q. Ab initio study of spectroscopic properties at anharmonic force fields of LiNH2. *J Mol Model* (2021) 27:1–13. doi: 10.1007/S00894-020-04641-9/TABLES/11

28. Barthet VJ, Petryk MWP, Siemens B. Rapid Nondestructive Analysis of Intact Canola Seeds Using a Handheld Near-Infrared Spectrometer. *J Am Oil Chem Soc* (2020) 97:577–589. doi: 10.1002/AOCS.12335

29. Som T, Karmakar B. Structure and properties of low-phonon antimony glasses and nano glass-ceramics in K2O–B2O3–Sb2O3 system. *J Non Cryst Solids* (2010) 356:987–999. doi: 10.1016/J.JNONCRYSOL.2010.01.026

30. Wei J, Zhao L, Peng S, Shi J, Liu Z, Wen W. Wettability of urea-doped TiO2 nanoparticles and their high electrorheological effects. *J Sol-Gel Sci Technol* (2008) 47:311–315. doi: 10.1007/S10971-008-1787-Z/TABLES/2

31. Cai CB, Tao YY, Wang B, Wen MQ, Yang HW, Cheng YJ. Investigating the adsorption process of isoamyl alcohol vapor onto silica gel with near-infrared process analytical technology. *Spectrosc Lett* (2014) 48:190–197. doi: 10.1080/00387010.2013.872668

32. Kakuda H, Okada T, Hasegawa T. Temperature-Induced Molecular Structural Changes of Linear Poly(ethylene imine) in Water Studied by Mid-Infrared and Near-Infrared Spectroscopies. *J Phys Chem B* (2009) 113:13910–13916. doi: 10.1021/JP9048204

33. Zhang YY, Wang C, Li G, Zang X, Yu Y, Hu HS, Yang J, Zhang W, Dai D, Wu G, et al. Infrared spectroscopic signature of the structural diversity of the water heptamer. *Cell Reports Phys Sci* (2022) 3:100748. doi: 10.1016/J.XCRP.2022.100748

34. Liu J, Liu R, Xu K. Accuracy of Noninvasive Glucose Sensing Based on Near-Infrared Spectroscopy. *Appl Spectrosc* (2015) 69:1313–1318. doi: 10.1366/14-07728

35. Saiga N, Hamada C, Ikeda J. Near infrared spectroscopy assessment of the glucose solution processed by ultrasonic cavitation. *Ultrasonics* (2006) 44:e101–e104. doi: 10.1016/J.ULTRAS.2006.06.029

36. Wiercigroch E, Szafraniec E, Czamara K, Pacia MZ, Majzner K, Kochan K, Kaczor A, Baranska M, Malek K. Raman and infrared spectroscopy of carbohydrates: A review. *Spectrochim Acta Part A Mol Biomol Spectrosc* (2017) 185:317–335. doi: 10.1016/J.SAA.2017.05.045

37. Chen S, Wang CY, Tsai CY, Yang IC, Luo SJ, Chuang YK. Fermentation quality evaluation of tea by estimating total catechins and theanine using near-infrared spectroscopy. *Vib Spectrosc* (2021) 115:103278. doi: 10.1016/J.VIBSPEC.2021.103278

38. Li H, Lv X, Wang J, Li J, Yang H, Qin Y. Quantitative determination of soybean meal content in compound feeds: Comparison of near-infrared spectroscopy and real-time PCR. *Anal Bioanal Chem* (2007) 389:2313–2322. doi: 10.1007/S00216-007-1624-1/FIGURES/6

39. Unger R, Braun U, Fankhänel J, Daum B, Arash B, Rolfes R. Molecular modelling of epoxy resin crosslinking experimentally validated by near-infrared spectroscopy. *Comput Mater Sci* (2019) 161:223–235. doi: 10.1016/J.COMMATSCI.2019.01.054

40. Hassanzadeh A, Murphy SP, Pethybridge SJ, van Aardt J. Growth Stage Classification and Harvest Scheduling of Snap Bean Using Hyperspectral Sensing: A Greenhouse Study. *Remote Sens 2020, Vol 12, Page 3809* (2020) 12:3809. doi: 10.3390/RS12223809

41. Wenz JJ. Influence of steroids on hydrogen bonds in membranes assessed by near infrared spectroscopy. *Biochim Biophys Acta - Biomembr* (2021) 1863:183553. doi: 10.1016/j.bbamem.2021.183553

42. Mastrapa RME, Moore MH, Hudson RL, Ferrante RL, Brown RH, Mastrapa RME, Moore MH, Hudson RL, Ferrante RL, Brown RH. Proton Irradiation of Crystalline Water Ice: Timescales for Amorphization in the Kuiper Belt. *DPS* (2005) 37:56.10. https://ui.adsabs.harvard.edu/abs/2005DPS....37.5610M/abstract [Accessed January 20, 2022]

43. Hu Y, Macfhionnghaile P, Caron V, Tajber L, Healy AM, Erxleben A, Mcardle P. Formation, Physical Stability, and Quantification of Process-Induced Disorder in Cryomilled Samples of a Model Polymorphic Drug. *J Pharm Sci* (2013) 102:93–103. doi: 10.1002/JPS.23338

44. Genkawa T, Watari M, Nishii T, Suzuki M, Ozaki Y. Two-dimensional heterospectral correlation analysis of water and liquid oleic acid using an online near-infrared/mid- Infrared dual-region spectrometer. *Appl Spectrosc* (2013) 67:724–730. doi: 10.1366/12-06871

45. Devasahayam S, Bandyopadhyay S, Hill DJT. Study of Victorian Brown Coal Dewatering by Super Absorbent Polymers using Attenuated Total Reflection Fourier Transform Infrared Spectroscopy. *Miner Process Extr Metall Rev* (2016) 37:220–226. doi: 10.1080/08827508.2016.1168417

46. Yoshino R, Hashimoto K, Omi T, Ishiuchi SI, Fujii M. Structure of 1-naphthol-water clusters studied by IR dip spectroscopy and ab initio molecular orbital calculations. *J Phys Chem A* (1998) 102:6227–6233. doi: 10.1021/jp9815006

47. Tang M, Gu W, Ma Q, Jie Li Y, Zhong C, Li S, Yin X, Huang RJ, He H, Wang X. Water adsorption and hygroscopic growth of six anemophilous pollen species: The effect of temperature. *Atmos Chem Phys* (2019) 19:2247–2258. doi: 10.5194/ACP-19-2247-2019

48. Bhattacherjee A, Wategaonkar S. Water bridges anchored by a C-H⋯O hydrogen bond: The role of weak interactions in molecular solvation. *Phys Chem Chem Phys* (2016) 18:27745–27749. doi: 10.1039/c6cp05469b

49. Honda R, Ryu M, Balčytis A, Vongsvivut J, Tobin MJ, Juodkazis S, Morikawa J. Paracetamol micro-structure analysis by optical mapping. *Appl Surf Sci* (2019) 473:127–132. doi: 10.1016/J.APSUSC.2018.12.121

50. Mohaček-Grošev V, Furić K, Vujnović V. Raman study of water deposited in solid argon matrix. *Spectrochim Acta Part A Mol Biomol Spectrosc* (2022) 269:120770. doi: 10.1016/J.SAA.2021.120770

51. Kim HD, Ishida H. A study on hydrogen-bonded network structure of polybenzoxazines. *J Phys Chem A* (2002) 106:3271–3280. doi: 10.1021/jp010606p

52. Park M, Shin I, Singh NJ, Kim KS. Eigen and Zundel forms of small protonated water clusters: Structures and infrared spectra. *J Phys Chem A* (2007) 111:10692–10702. doi: 10.1021/jp073912x

53. Shekaari H, Modarress H. Excess thermodynamic properties calculations for alcohols in inert solvents based on Fourier transform infrared spectroscopy measurements. *J Sci Islam Repub Iran* (2003) 14:133–140.

54. Fengel D. Influence of Water on the OH Valency Range in Deconvoluted FTIR Spectra of Cellulose. *Holzforschung* (1993) 47:103–108. doi: 10.1515/HFSG.1993.47.2.103/MACHINEREADABLECITATION/RIS

55. Paladini G, Venuti V, Crupi V, Majolino D, Fiorati A, Punta C. 2D Correlation Spectroscopy (2DCoS) Analysis of Temperature-Dependent FTIR-ATR Spectra in Branched Polyethyleneimine/TEMPO-Oxidized Cellulose Nano-Fiber Xerogels. *Polym 2021, Vol 13, Page 528* (2021) 13:528. doi: 10.3390/POLYM13040528

56. Frost RL, Erickson KL, Čejka J, Reddy BJ. A Raman spectroscopic study of the uranyl sulphate mineral johannite. *Spectrochim Acta Part A Mol Biomol Spectrosc* (2005) 61:2702–2707. doi: 10.1016/J.SAA.2004.10.013

**
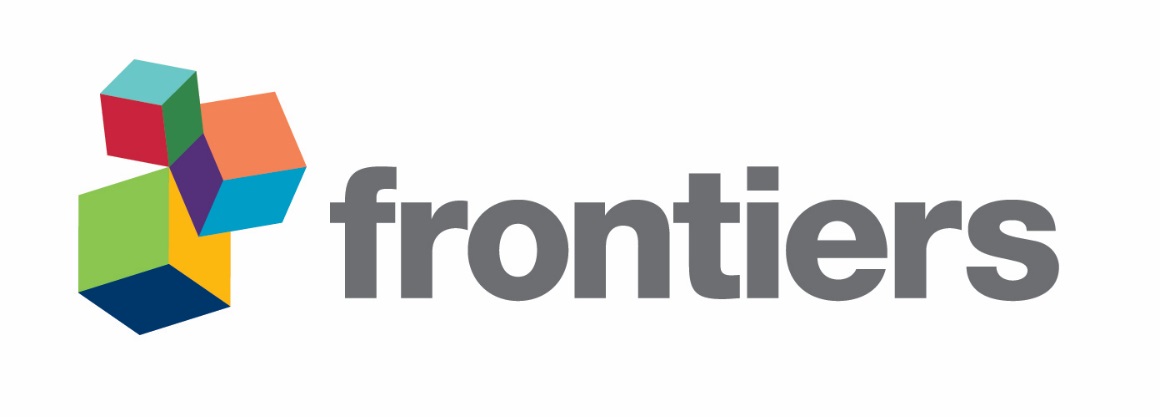
**
